# The interaction between language and working memory: a systematic review of fMRI studies in the past two decades

**DOI:** 10.3934/Neuroscience.2021001

**Published:** 2020-11-16

**Authors:** Zoha Deldar, Carlos Gevers-Montoro, Ali Khatibi, Ladan Ghazi-Saidi

**Affiliations:** 1 Department of Anatomy, Université du Québec à Trois-Rivières, Trois-Rivières, QC, Canada; 2 Madrid College of Chiropractic, Real Centro Universitario María Cristina, San Lorenzo de El Escorial, Madrid, Spain; 3 Centre of Precision Rehabilitation for Spinal Pain, University of Birmingham, Birmingham, UK; 4 Centre for Human Brain Health, University of Birmingham, Birmingham, UK; 5 Language and Cognition Laboratory, Department of Communication Disorders, College of Education, University of Nebraska at Kearney, USA

**Keywords:** language, working memory, neuroimaging, fMRI, neural networks, cognitive processing, neurocognition, verbal working memory, interaction

## Abstract

Language processing involves other cognitive domains, including Working Memory (WM). Much detail about the neural correlates of language and WM interaction remains unclear. This review summarizes the evidence for the interaction between WM and language obtained via functional Magnetic Resonance Imaging (fMRI) in the past two decades. The search was limited to PubMed, Google Scholar, Science direct and Neurosynth for working memory, language, fMRI, neuroimaging, cognition, attention, network, connectome keywords. The exclusion criteria consisted of studies including children, older adults, bilingual or multilingual population, clinical cases, music, sign language, speech, motor processing, review papers, meta-analyses, electroencephalography/event-related potential, and positron emission tomography. A total of 20 articles were included and discussed in four categories: language comprehension, language production, syntax, and networks. Studies on neural correlates of WM and language interaction are rare. Language tasks that involve WM activate common neural systems. Activated areas can be associated with cognitive concepts proposed by Baddeley and Hitch (1974), including the phonological loop of WM (mainly Broca and Wernicke's areas), other prefrontal cortex and right hemispheric regions linked to the visuospatial sketchpad. There is a clear, dynamic interaction between language and WM, reflected in the involvement of subcortical structures, particularly the basal ganglia (caudate), and of widespread right hemispheric regions. WM involvement is levered by cognitive demand in response to task complexity. High WM capacity readers draw upon buffer memory systems in midline cortical areas to decrease the WM demands for efficiency. Different dynamic networks are involved in WM and language interaction in response to the task in hand for an ultimate brain function efficiency, modulated by language modality and attention.

## 1. Introduction

This manuscript aims to provide a review of the interaction between language and Working Memory (WM) by reviewing the functional MRI studies published between 2000 and 2020. Language is the most essential and prominent mode of communication in humans, and WM is considered one of the main functions of the cognitive domain showing the most interaction with language. Functional MRI provides excellent information on the spatial localization of brain functions and the underlying neurocognitive processes of language and WM.

Understanding the cognitive and neural processes of language and its interaction with other higher cognitive domains is essential for three reasons. First, it will help us gain a better understanding of how a healthy human brain functions. Secondly, this will help us develop better strategies to learn and teach a new language. Finally, and most importantly, such knowledge can help us better define pathological signs and symptoms, develop better and more efficient assessment tools, and find more efficient and successful intervention techniques. A more detailed account of the interaction between language and WM is given in the following section. We will first provide a brief overview of language definition and processes, followed by WM definition and models, and a brief description of functional neuroimaging. We will then focus on the interaction between language and WM through the lens of fMRI studies. We will end the review by enunciating the limitations and future directions in this field.

## 2. Language

Language or verbal communication is the most effective way of communication. Language has different components (context, syntax, semantics, morphology, phonology and pragmatics) and can be used in different modalities (speaking, listening, reading and writing). Language is studied from different perspectives: linguistics, psycholinguistics, and neurolinguistics. Neurocognitive studies discuss language by merging the two latter and basing the roots in linguistics.

From the linguistic viewpoint, language can be divided into semantics, syntax and phonology. Semantics is the meaning of language at all levels; words, sentences, and the relationship among linguistic elements, including phonemes, morphemes, lexemes, syntax, context and pragmatics [1]–[4]. The smallest unit of language that can be used independently for effective communication is the word. Words that are associated with one another based on their meanings fall into different categories. Superordinate, coordinates and subordinates (category membership based on the degree of relatedness and inclusion), synonyms (words sharing the same meaning), antonyms (words with opposite meanings) and hyponyms (words in the same category) are such examples [5]. Thus, words belong to a network. Words can have different meanings in different contexts (e.g., literal vs. metaphor or allegory) [3],[6]. This can sometimes cause ambiguity. Other sources of ambiguity are homophones (words with the same sound but different meanings), and homographs (words with identical spellings but different meanings). Processing such words engage WM [7],[8].

Phonology studies the mental aspect of sound systems and patterns [9],[10]. Phonetics is studying the characteristics of the physical articulation of speech sounds [11]. Each given language has its specific phonological rules. Speakers of a language have knowledge about the phonemics and phonetics of the words in their language and the relationship between them [9],[12]. To produce a word, the speaker needs to associate the semantics with the phonetics and the phonemics. WM allows this association that is necessary for articulation. Articulation is the production of sounds by manipulating speech organs [3],[6]. The phonetic and phonological knowledge is processed in the WM, where they interact with the executive function system (i.e., planning, programming, selection/inhibition), projecting to the motor speech system for execution of a successful articulation [3],[6]. In acquired or developmental speech and language disorders, one or more components of this complex and interactive system fail.

Cognitively, language processing is typically discussed separately for comprehension or perception (understanding) and production or expression (generating). Classically, cognitive processing of language comprehension and production are affiliated to specific areas of the human brain (i.e., Broca's area and Wernicke's area). Broca's area is known to be involved in both production and comprehension [3],[6]. It overlaps parts of the left inferior frontal gyrus (IFG); Brodmann areas (BA) 44 and 45. BA 45 engages in language interpretation as well as motor planning and motor programming of language production [3],[6]. Interacting with the motor cortex (BA 6), BA 44 initiates and coordinates the speech. Broca's area also processes word retrieval and spelling. The homologue of the Broca's area in the right hemisphere processes intonation and prosody and interprets the emotion behind the language content. Wernicke's area is located within the left superior temporal gyrus (STG), including BA 20, 21 and 38 and some parietal areas [13],[14]. Wernicke's area processes language comprehension by attaching meaning to auditory information (processed in BA 41 ad 42). We now know that many brain areas are engaged in language production and comprehension [15]. The initiation and execution of speech are processed in the left putamen, SMA (Supplementary Motor Area), pre-SMA, and motor cortex [16],[17]. Articulation is planned in the left anterior insula; words involving the left middle frontal cortex are retrieved, and involuntary response reactions are suppressed in the anterior cingulate and bilateral head of the caudate nuclei. Bilateral STG or Heschl's gyrus in the temporal lobes are important for the perception of auditory stimuli [16],[17]. Comprehension of sentences is associated with activations in the bilateral superior temporal sulci, and meaningful speech activates the middle and inferior temporal cortex [15]–[17]. The left angular gyrus and the pars orbitalis are reported to be involved in semantic retrieval [15]–[19]. Phonological processing of written alphabetic words is associated with the cortical areas of three neural networks: the ventral prefrontal system involving superior portions of the left IFG; the left dorsal temporoparietal system, including the mid-STG and the ventral aspect of the inferior parietal cortex (supramarginal region), and the left ventral occipitotemporal system [20].

Language production and comprehension occur through interaction with other cognitive domains [7]–[15] processed by different brain areas and linked by functional and anatomical circuits/networks [21]–[23]. Language is a functional system rather than an anatomical one [24] and depending on the task's nature and complexity, as well as proficiency, can involve more or fewer brain areas [25]–[27]. Therefore, it is not easy to determine what brain areas are engaged in processing language and how the language system interacts with other cognitive domains.

Language has been studied using different tasks and in different modalities: speech production [28]; written production [29],[30]; reading comprehension [28],[31]; and speech listening [32]. Tasks used to study language processing include semantic judgment, phonological judgment, word recognition, word categorization, verbal fluency, word and nonword repetition, picture naming as well as translation in bilingual speakers. The most commonly used task in neuroimaging language studies seems to be picture-naming [33]–[40].

Language processing has been associated with six connectivity pathways [41]. First, the ventral semantic stream, which connects the inferior occipitofrontal fasciculus to the posterior temporal regions and the dorsolateral prefrontal areas bidirectionally. In the dorsal phonological stream, the arcuate fasciculus (AF) connects Broca's and Wernicke's areas through connections between the posterosuperior temporal cortex and the posterior part of the inferior frontal cortex. The speech perception pathway (third pathway), connects the posterior temporal regions and the supramarginal gyrus. The articulatory loop makes up the fourth network. This loop connects the supramarginal gyrus and the inferior frontal cortex and sub-serves verbal WM. The cortico-striatal loop supports the interaction between language and executive function (i.e., selection, inhibition and programming). This loop connects the fronto-mesial structures to the head of the caudate nucleus (fifth pathway). The sixth pathway carries the speech production by linking the anterior insula and ventral premotor cortex to the primary sensorimotor area of the mouth [41].

## 3. Working memory

WM is involved in all domains of higher cognition including memory, executive function, visuospatial processing, attention and language [42],[43]. Complex cognitive processes, including language comprehension or production, reasoning, decision-making, and top-down attentional processes, are some examples [43]. WM maintains and processes information actively for a short time by prioritizing the maintenance of task-relevant information in the face of task-irrelevant information [42]–[46].

The literature is not convergent on whether WM is an independent entity or takes part in another cognitive system [47],[48]. Some authors argue that WM is one of several executive functions controlling cognitive performance [49],[50]. Others hypothesize that it is part of the memory system [51],[52]. WM can engage in the three distinct memory processes: encoding, maintaining and manipulating information [53]–[55]. In older literature, WM has been sometimes used interchangeably with short-term memory [56]. The concept of WM is now distinguished from the concept of short-term memory [57]. It has been clarified that while short-term memory exclusively entails the storage of information, WM includes both storage and manipulation of information [42],[53],[58].

Several models have defined WM as a specific cognitive process, while others have described its corresponding anatomical structures [58]. A comprehensive neurocognitive model is yet to be proposed. The most influential WM model is a cognitive multicomponent model proposed by Baddeley and Hitch [44]. This model introduces four central mechanisms within WM function: the phonological loop, the visuospatial sketchpad loop, the central executive, and the episodic buffer [42],[44]. The phonological loop stores sound (phonological information). Visual and spatial information about stimuli (e.g., shape, color, and location) are maintained in the visuospatial sketchpad loop. The central executive controls information processing in the phonological and visuospatial sketchpad loops. The central executive is responsible for updating and manipulating information, directing attention to targets, inhibiting the processing of irrelevant information, and controlling cognitive processes during multitasking. Baddeley and Hitch proposed that the central executive is responsible for controlling attentional processes instead of being simply a memory storage. This makes it different from the phonological and the visuospatial sketchpad loops, whose jobs are limited to storing information. Indeed, the central executive, similarly to executive attention, selects relevant stimuli and ignores irrelevant stimuli [42],[44],[47],[53],[59]–[61]. The episodic buffer-which was added recently as the fourth element of the model-maintains multimodal representations of information like semantic information. Additionally, it integrates information from different loops to form a coherent whole, combines information, and links WM to long-term memory [42],[44],[53].

There are several other well-articulated models of WM that may differ in fundamental ways in conceptualizing WM function [58]. Although these models may seem to be entirely different from each other, these distinctions are mainly based on the distinctive terminology and their focused research area. These models consider a link between WM and long-term memory and emphasize the function of the central executive as a control system to monitor, manipulate information and inhibit distractors. However, so far, behavioral findings alone have been insufficient to adjudicate these theories. Neuroimaging methods, on the other hand, have the potential to localize the neural substrates of WM, and more importantly, to provide a novel set of constraints that may help in evaluating the adequacy of alternative models. In this regard, neuroimaging studies can play a critical role in advancing theoretical models.

An array of neuroimaging studies has attempted to unveil brain activity underlying the performance of WM tasks using different kinds of stimuli. The study of verbal and visuospatial WM has provided an insight into independent WM systems with distinct neurological representations, consistent with the multicomponent model of WM [62]. It has been suggested that the phonological loop is supported by Broca's and Wernicke's areas, which are involved in the processing of verbal and acoustic information [62]. In particular, the inferior frontal cortex, including Broca's area, supported rehearsing information during storage [63]. On the other hand, the visuospatial sketchpad is represented by right hemispheric areas [62], particularly the right posterior parietal cortex [64].

The WM function is partially supported by the executive function. The frontoparietal cortex, is involved in cognitive control [65],[66]. The frontoparietal system is involved in engaging attention towards relevant external stimuli [67]. The frontoparietal network has been extensively implicated as the main WM neural network [68]. This network involves mainly the dorsolateral prefrontal cortex (DLPFC), which supports executive control processes [63],[69],[70] and maintaining and manipulating information [71]–[74]; the anterior cingulate cortex (ACC) as the “attention-controller” [71]; and the parietal cortex as a “workspace” for sensory processing [68],[75],[76]. Other subcortical structures have been involved with WM functions, mainly the basal ganglia during verbal WM and the thalamus during maintaining of information (i.e., the medial nuclei) [72] or directing attention towards goal-relevant items (i.e., the pulvinar) [77],[78]. In addition, WM is supported by the default-mode network (DMN), known to be active during the resting state. The DMN has been related to internally directed thoughts [79]. Hence, the WM function is supported by two systems with opposite influences on the cognitive state: while the frontoparietal network directs outward attention, the DMN directs cognition internally [67],[80].

A key feature of the WM system is its capacity limitation for the amount of information that it can maintain at once [53],[81],[82]. Cowan's embedded-processes model of WM suggests that WM has a capacity limit of approximately 3 or 4 simple items [83]. Holding information in WM depends on how many items can be grouped under units (i.e., coupling items into new units that can be rapidly recalled), or “chunks” (i.e., building more extensive collections of items by using what we already know). Grouping and chunking help to maintain information in WM efficiently by decreasing the number of items that must be memorized. However, complex items may reduce WM performance as those complex items require additional resources in order to encode information in detail [84],[85]. Taken together, WM performance depends on the nature of the WM task and the demands related to those specific tasks. Grouping and chunking information may increase WM performance, while complex items may reduce it.

Engle and colleagues have proposed that an executive attention control mechanism can affect WM capacity and WM performance [60]. This executive attention control mechanism is mediated by activity in the prefrontal cortex (including the DLPFC), where goal-relevant information can be actively maintained even in the presence of distractors [60],[86]. Likewise, Unsworth and colleagues have proposed that individual differences in WM performance can be associated with differences in three different mechanisms: 1) attentional control, which is the ability to maintain relevant information despite the presence of distraction; 2) the number of items that can be stored according to the capacity of WM; or 3) the ability to retrieve information from long-term memory and bring it into the focus of attention [87]. These models attempt to explain the WM concept and individual differences in WM performance [86].

## 4. Functional imaging of the human nervous system

Although models of WM often make little contact with models of language processing, we will argue below that neuroimaging data can be used to highlight and understand important points of connection that have been generally overlooked (for related discussion, see Smith & Geva [88]). Functional Magnetic Resonance Imaging (fMRI) has become the choice method for many studies interested in investigating the nervous system's performance in humans. It allows an online examination of the nervous system's function with a reasonable temporal and spatial resolution. Even though fMRI is only an indirect measure of the neural functioning, its contribution to our understanding of the organization of different areas of the brain [89] and the intra-area organization of signal processing in the central nervous system [90] is remarkable.

In the study of language and WM processes, fMRI had a significant influence on our understanding of how information is encoded in the nervous system, how language disorders influence the function of specific brain areas, and the connection between those areas and the rest of the brain [91]. It also helps us understand the cerebral mechanisms of learning a language [91]. Besides the lesion studies, fMRI studies are among the most informative, allowing us to know which brain regions contribute to cognitive processes involved in linguistic tasks [92]. From a clinical perspective, this information is beneficial in helping us to determine the extent of an injury in a patient with cerebral damage. fMRI has become a measure for presurgical assessment of patients undergoing brain surgery to minimize the chance of damage to areas involved in language and motor tasks [93].

However, fMRI is not the only method used to study language processing in the human brain. Many studies used other neuroimaging techniques such as Event-Related potentials [94] and functional near infra-red spectroscopy [95] to investigate the brain's function during language and cognitive mechanisms. Each of these methods has its advantages and disadvantages. ERP benefits from higher temporal resolution but a lower spatial resolution, while fMRI is known for a better spatial resolution at the cost of lower temporal resolution. This advantage makes fMRI a favorite method when it comes to the study of the mechanisms that involve subcortical areas of the brain and thus for those interested in the study of cognitive processes involved in language [96]. The low temporal resolution does not allow for the study of cognitive mechanisms at earlier stages of processing. Accordingly, the study of WM's involvement in linguistic processes becomes advantageous over other mechanisms such as attention.

## 5. Interaction between language and working memory as assessed by fMRI

**Figure 1.**
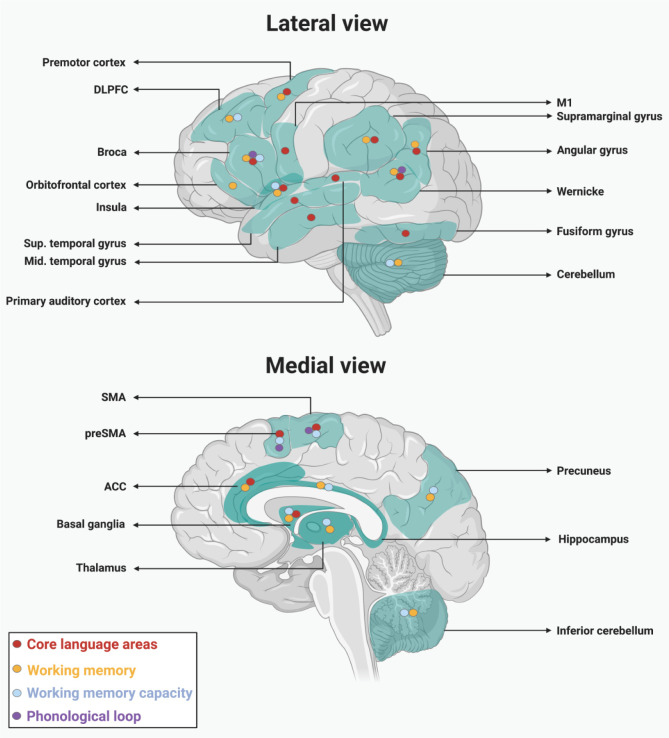
Brain areas associated with the interaction between language and working memory.

To review the studies on the interaction of language and WM, we searched PubMed, Google Scholar, Science direct and Neurosynth. The search terms were limited to working memory, language, fMRI, neuroimaging, cognition, attention, network, connectome. We also searched reference lists of papers that were identified as particularly relevant from the PubMed search. In Neurosynth, once, we limited our search to “Language” and overlapped the keyword “Working Memory”. Then, we limited the search to “Working Memory”, and overlay the search with “Language”. Together, 147 papers were identified with the keywords in the title or the abstract. The research team, individually, selected a shortlist based on whether the study included a WM task and met the inclusion (as described above) and exclusion criteria. The exclusion criteria consisted of studies including children, older adults, bilingual or multilingual population, clinical cases, music, sign language, speech, motor processing, review papers, meta-analyses, electroencephalography/event-related potential (EEG/ERP), and positron emission tomography (PET) studies. An excel sheet was used for all research team members to subjectively (their interpretation) include or exclude papers based on relevance. Papers with 3/4 inclusion-vote were included in the review. A total of 20 articles were included. We categorized these papers based on their core topic into four main categories: language comprehension, language production, syntax, and network. Table 1 summarizes these 20 studies. Precisely, Table 1, includes the aims and some basic methodological aspects, including sample sizes and tasks. Further, the technical aspects related to fMRI methodology are detailed in Table S1 as supplementary material. Additionally, Figure 1 depicts the main brain activations reflecting language-WM interaction, based on the 20 papers that met the inclusion criteria for this review. The synthesis of each topic was composited and summarized, which will follow.

This figure displays the main brain regions that have been reported to be involved in tasks reflecting the interaction between language and working memory. Multiple brain regions include: Broca's area; Wernicke's area; ACC = Anterior Cingulate Cortex; Insula; M1 = Primary Motor Cortex; SMA = Supplementary Motor Area; preSMA = pre-Supplementary Motor Area; Thalamus; Premotor Cortex; DLPFC = Dorsolateral Prefrontal Cortex; Orbitofrontal Cortex; Sup. temporal gyrus = Superior temporal gyrus; Mid. temporal gyrus = Middle. temporal gyrus; Primary Auditory Cortex; Supramarginal Gyrus; Angular Gyrus; Fusiform Gyrus; (Inferior) Cerebellum; Hippocampus; Basal Ganglia; Precuneus. Four categories, including core language areas, phonological loop, working memory and working memory capacity are associated with each area according to the specific role during this interaction. The regions are all projected on a mid-sagittal and lateral view of the brain. *Created with BioRender.com*.

### 5.1. Language comprehension

Language comprehension is a specialized higher function that involves various processes, which convert external cues (mostly auditory and visual stimuli) into meaningful messages for the receiver. Regardless of the modality of presentation, language comprehension entails the processing of the sensory information (auditory or visual), phonological or orthographic processing, as well as the meaning (semantics) associated with the phonological form. Comprehending language and overcoming barriers linked to complex linguistic stimuli rely on the interaction between different cognitive domains, including memory, executive functions, attention, and WM. Neural correlates of multiple brain functions have been studied in the past decades. Specifically, functional MRI studies have used a variety of cognitive and/or linguistic tasks that could potentially reflect the interaction between language comprehension and WM (Table 1) [16],[97]–[99].

Language comprehension is not only dependent on context, but also on the receiver's WM capacity. The context in which language is presented is an essential element to its comprehension. During language comprehension tasks, altering the semantic and syntactic context in which words are presented can be used to increase the level of complexity. Individual WM capacity also influences the degree of comprehension. WM capacity refers to individual differences in construct ability, reflecting the size (limit) of of their WM. By manipulating the linguistic complexity level, WM demands can be adjusted. Linguistic complexity and WM demands correlate negatively with WM capacity [98],[99]. In order to assess for different WM demands and individual differences, performance and brain activity are examined during tasks manipulating linguistic complexity. Reading span tasks, altering syntactic structure, or context ambiguity are some of the tools utilized to manipulate linguistic complexity. The studies reviewed below provide evidence on how language modality and different semantic and syntactic contexts can modulate the levels of task complexity. Task complexity affects the processing of language comprehension by modulating WM demands. In addition, we will discuss the impact of individual differences on WM capacity and, therefore, on cognitive processes of language comprehension.

**Table 1.** A summary of the aims and methodological approaches (sample size and tasks used to manipulate language and working memory (WM) of the 20 studies included in this review.

Authors (year)	Aim of studies	Participants		Tasks	
		Experimental group	Control group	Language	Working memory
Buchweitz et al. (2009) [16]	Brain activation for listening and reading comprehension processes & individual differences in WM capacity	N = 12	N/A	Auditory and visual sentences' comprehension	Reading span task: rapid serial visual presentation format (RSVP)
Moore et al. (2013) [72]	Study of a model of verbal WM with specific focus on basal ganglia. Potential differences in neural function across the complete process of verbal WM	N = 14	N/A	Identification of semantic relationship between 2 words in a pair	Verbal WM task
Buchweitz et al. (2012) [97]	Brain activation in dual task vs single-message comprehension & individual differences in WM capacity	N = 12	N/A	Single vs. two concurrent spoken sentences comprehension	Reading span task: rapid serial visual presentation format (RSVP)
Fiebach et al. (2004) [98]	Neural correlates of syntactic ambiguity & individual differences in WM capacity.	N = 15 (divided in 2 groups)	N/A	Temporarily ambiguous sentences	Reading span task
Mason & Just (2007) [99]	Changes to cortical networks in processing ambiguity & individual differences in WM capacity.	N = 12	N/A	Lexical ambiguity task	Reading span task
Rudner et al. (2013) [102]	Neural representation of language modality specificity (semantic, phonological and orthographic) processing in WM	N = 20 (hearing non-signers)	N = 11 (deaf signers)	2-back task conditions according to Semantic, Phonological, Orthographic and Colour baseline criteria	2-back task: 4 different linguistic conditions
Rudner et al. (2005) [103]	Neural correlates of mentally reversing spoken items (and comparison with a rhyme judgment task)	N = 12	N/A	Auditory word reversal task	Rhyme judgment task
Newman et al. (2013) [104]	Impact of WM capacity on sentence comprehension, task activation and connectivity between language and WM-related regions	N = 50	N/A	Sentence comprehension task, varying syntactic complexity	Reading Span task
Wallentin et al. (2006) [110]	Neural correlates of efficient involvement of WM systems during language comprehension	N = 21	N/A	Spoken sentences comprehension and verbally-cued recall	Spatial and nonspatial recall of image elements
Marvel & Desmond (2012) [112]	Neural correlates of inner speech processes related to manipulating versus storing verbal content during WM	N = 16	N/A	Letter recognition and recall (using a probe) task	Verbal WM task: Sternberg task
McGettigan et al. (2011) [113]	Neural correlates of sublexical structure in phonological WM	N = 17 Experiment 1 N = 15 Experiment 2	N/A	Experiment 1: Covert rehearsal task Experiment 2: Separate passive listening experiment	Digit span task
Powell et al. (2012) [114]	The effect of handedness, language & spatial laterality on verbal comprehension, WM and perceptual organization.	N = 42 (right-handed individuals)	N = 40 (left-handed individuals)	Verbal fluency word generation task	The Wechsler Adult Intelligence Scale
Sahin et al. (2006) [115]	Neural substrates of grammar different from WM, semantics, phonology, or lexical processing. Brain regions that are active in inflectional morphology	N = 18	N/A	Cued covert word generation task	Cued language production (higher WM load) was compared to reading (no load)
Meyer et al. (2012) [116]	Spatiotemporal neuronal dynamics of argument retrieval and reordering processes	N = 14	N/A	Syntactic comprehension and construction task	Task used sentence stimuli that required reordering and retrieving arguments
Bonhage et al. (2014) [117]	Brain mechanisms underlying the sentence superiority effect during encoding and maintenance in WM	N = 18	N/A	Maintenance of sentence structured fragments vs. unstructured word collections	Task manipulated WM load and articulatory suppression during maintenance
Makuuchi & Friederici (2013) [121]	The dynamics of the neural network supporting processing sentences with varying syntactic complexity	N = 22	N/A	Sentence comprehension with manipulation of syntactic complexity	Reading Span task
Newman et al. (2002) [125]	Differences in timing of WM network responses during the presentation of two types (loads) of verbal problems	N = 14	N/A	Written sentences comprehension task	Verbal WM task: two types of verbal problems (early/low load vs. late/high load).
Cooke et al. (2006) [127]	Neural basis for processing different aspects of a sentence depending on WM demands associated with a particular grammatical feature	N = 15	N/A	Sentence-processing task related to its structure-building component	WM resource demands manipulated during sentence processing
Tomasi & Volkow (2020) [128]	The ability to predict reading accuracy and single-word comprehension scores from rest and task fMRI data. The effect of motion in the prediction of language from fMRI data	N = 424	N/A	Semantic comprehension and oral reading recognition tasks	n-back task: 4 different picture categories faces, places, tools, body parts), presented in 8 separate blocks
Mineroff et al. (2018) [134]	The relationship among the language, multiple demand, and default-mode networks.	N = 60	N/A	Language comprehension task (words vs. nonwords)	Spatial working WM task with two different loads

#### 5.1.1. Language complexity: speech modalities

Language modality is the most basic contextual variable that can influence comprehension and processing. Comparisons of listening and reading comprehension tasks suggest that the initial processing step elicits activity in modality-sensitive areas [16]. Thus, printed language engages the left inferior occipital lobe to process visual stimuli, including the left fusiform gyrus (associated with word recognition). On the contrary, listening comprehension induces activation of the STG, which includes the primary auditory cortex and Wernicke's area [16]. In addition, a left-lateralized network, including the left IFG and middle temporal gyrus (MTG) is activated independently of language modality. Oral language results in more overall brain activation, particularly in the right hemisphere.

It has been argued that this reflects a higher level of WM engagement to maintain and store information during a listening task [16]. At the word level, language modality includes phonology, orthography and semantics. Comparing phonological, orthographic and semantics processing, a 2-back task^1^ [100],[101] was used to increase WM demands for these specific language processing conditions [102]. The 2-back task was modified so that participants were asked to match a current picture with pictures presented two steps back based on semantic, phonological or orthographic similarity criteria [102]. This WM task showed evidence for the involvement of the right hemisphere in orthographic and semantic processing (as shown during disambiguation). The phonological condition resulted in more activation of the posterior portion of the left IFG (BA 45), which could be expected considering its role in phonological rehearsal. The orthographic condition recruited the left MTG and a right-lateralized network of posterior cortical areas. The semantic condition induced more activation in the right superior frontal gyrus (BA 10 in the prefrontal cortex) [102]. Activation of right hemispheric regions could indicate that increasing WM demands during orthographic and semantic processing requires the implication of additional neural networks and cognitive resources.

#### 5.1.2. Language complexity: semantic ambiguities

Semantic interpretation is fundamental to language comprehension. For an accurate semantic interpretation, considering context is crucial. A linguistic concept related to semantic interpretation that can increase the linguistic complexity of the context and therefore increase cognitive demand, is ambiguity. Words and even full sentences with ambiguous meanings become more explicit when the contextual information has been accounted for. Typically, it is essential for the listener/reader to maintain alternative meanings of words [99] or longer speech formats [98] available for accurate comprehension. Both strategies require the engagement of WM. Lexical or sentence ambiguity leads to either an early or a late selection of meaning. Once a meaning is selected, possibly multiple mental imagery systems come into play to generate comprehension [103]. Ambiguous words and sentences have been used to investigate the neural correlates of semantic interpretation and the role that context plays in language comprehension. This process also requires WM involvement. Processing ambiguity involves the left IFG (including BA 44/45), and the superior frontal cortex at an early interpretation of the ambiguous word. This is followed by the involvement of the right IFG and the insulae to suppress the incorrect interpretation when the word has been disambiguated [98],[99]. It has also been suggested that the basal ganglia are engaged in semantic selection and suppression of the appropriate and inappropriate meanings of an ambiguous word or sentence [72],[98],[104].

#### 5.1.3. Language complexity: reading span tasks

A linguistic task typically used to measure the interaction between WM and language comprehension is the Reading Span task^2^. Reading span tasks recruit WM in order to maintain multiple meanings during the reading task [98],[99]. Processing syntactic complexity (e.g., object-relative sentences) further engage WM systems. Both syntactic demands and reading span are associated with WM capacities and correlate heavily with right hemisphere activation [98],[99],[104]. This is in line with the general function of the right hemisphere in the comprehension of complex and long discourses.

Reading span tasks can also manipulate language complexity by introducing complex syntax. Syntactically complex sentences (e.g., object-relative sentences) during reading span tasks provide a means to evaluate the impact of increasing WM demands [104]. Processing syntactically complex sentences increase activation in the bilateral caudate. Increased activity in the basal ganglia during the processing of syntactically complex sentences could reflect efforts to maintain different potential interpretations [104]. Processing complex syntax also involves the DLPFC, the precuneus and the inferior parietal cortex, all of which reflect a higher WM demand [103],[104].

Dual tasking further increases WM demands [97]. The interaction between WM and language comprehension has been reported when participants were presented with either a single or two concurrent spoken sentences [97]. Both tasks individually induced activity in core language areas (left IFG, STG and MTG). During the dual-task, the same regions and surrounding neuronal pools became further active, accompanied by the recruitment of their counterparts in the right hemisphere [97]. This pattern is consistent with the activation of the right hemisphere when complexity increases. Interestingly, reading span scores, although positively correlated with comprehension accuracy in the single tasks was not able to predict performance during the dual-tasks [97]. This is compatible with the dual-task engaging different brain areas (mostly in the right temporal lobe) when compared to the single task.

#### 5.1.4. Individual differences in WM capacity

Regarding individual differences in WM capacity and its effect on language comprehension, Buchweitz et al. demonstrated that reading span scores were correlated with recruitment of the left versus right hemisphere voxels [16]. Lower WM capacity readers (but not listeners) adapted by using more right hemispheric areas [16]. Additionally, low capacity readers also engaged more the left DLPFC.

Other studies have shown that lower WM capacity readers demonstrate right-lateralized activation of the superior temporal and prefrontal cortices (right IFG) [99]. The latter is associated with higher executive control demand. Activation of this network is also seen with increased attention demands towards a stimulus to maintain previous information active in WM [105],[106]. In addition, low span readers recruit the right basal ganglia and thalamus [98]. Activation of the basal ganglia reflects WM efforts at maintaining alternative meanings available. The function of the basal ganglia has been reported specific to verbal WM processes from encoding to retrieval, as well as suppressing distractors, which could occur in coordination with the left DLPFC [72]. The role of the basal ganglia in processing WM is well known [107],[108]. The basal ganglia link the frontal and parietal cortices, which allows the interaction between attentional control and WM, therefore contributing to individual differences in WM capacity [109].

Similarly, activation of the caudate was found to correlate with the performance of a verbally-cued recall task [110]. However, this was not the case when the linguistic cue was spatial; in that case, performance correlated with precuneus activation. This activation pattern is particularly reported in better performers who seem to down-regulate amygdalar activation [110]. Conversely, activity in the amygdala was reported to be negatively correlated with cognitive demand and performance. Activation of the amygdala may reflect emotional regulation during highly demanding tasks or emotionally loaded contexts [110].

Assessing higher individual WM capacities by using reading span tasks during disambiguation showed inconsistent results. Mason and Just reported a positive correlation between WM capacity and bilateral insular activity [99]. Conversely, Fiebach et al. observed activation with primary visual cortical areas and an area in the left planum temporale linked with high reading span capacity [98]. During complex syntactics, connectivity between the posterior cingulate cortex (PCC), the left IFG and the inferior parietal cortex were correlated with high WM capacity [104]. This was interpreted as high capacity readers relying on episodic memory systems during semantic processing, to generate a more elaborate representation of the sentence's meaning [104]. Overall, besides involving phonological WM loops, individuals with high WM capacity tend to recruit executive processes that are not language-specific in the planum temporale and widespread medial cortical areas, including episodic buffers.

Overall, the literature on the interaction of language comprehension and WM suggests that increasing linguistic complexity (e.g., semantic ambiguity, syntactic complexity or dual-tasking) can increase the WM load. During language comprehension, the WM load is reflected by the initial involvement of the temporal and occipital cortices, most likely reflecting lower-level processing. The left IFG is consistently found to be active during various phases of processing, particularly with phonological processing demands. However, depending on the context, levels of complexity or receiver's WM capacity, other higher brain areas and mechanisms tend to be engaged. In low WM capacity individuals and high language complexity, a shift has been observed towards right-hemispheric processing. This could reflect an adaptation during complex language towards visuospatial WM functions, instead of classic phonological loops. Additionally, these individuals rely more on the basal ganglia in the selection process for alternate meanings or interpretations. Beyond phonological loop circuits, high WM capacity individuals may also depend on the recruitment of distinct brain regions, possibly engaging the episodic memory network (medial prefrontal cortex, PCC, insula). However, the role played by these regions is still unclear.

### 5.2. Language production

Language production is a multilayered process and involves speech as well as cognitive processes, including semantics, executive functions (decision-making, planning, organizing), memory, attention and WM. Speech is a motor linguistic response that begins with a mental concept and results from a complex cognitive process. Language production, like language comprehension, is strongly influenced by context. Precisely, language production is initiated by mental planning, which results from environmental, cognitive and emotional contextual factors. Once a message is selected, it needs to be encoded into words with an intended meaning (semantics), and grammatically encoded to provide critical contextual information (i.e., syntactic). This provides further meaning to the words chosen [111]. This linguistic form goes through a final motor encoding phase to transform the mental process into sounds or written spelling. Every step of language production, from retrieving the mental concept, associating it to the semantic form to planning, programming and execution of the phonological form, requires WM. However, only a few studies have directly addressed the neural correlates of language production and WM. It is important to note the inherent difficulties of assessing language production using fMRI without generating artifacts. For this reason, some studies have focused on the mental representation immediately preceding motor speech.

In this regard, it has been suggested that verbal WM capacity may be enhanced by an inner speech mechanism during the translation of mental representation into motor speech regions [112]. The implication of a so-called inner speech network relying mostly on premotor cortical and subcortical areas (the left IFG, premotor cortex, SMA, pre-SMA, anterior insula and the cerebelli) was investigated during higher verbal WM demands. Task difficulty resulted in the activation of the right DLPFC and the left inferior cerebellum. These regions have been both considered to be part of classic WM loops. The results suggest that the inner motor representations are further activated to share the load with classic WM systems [112]. For a schematic illustration of the left IFG, premotor cortex, SMA, pre-SMA, anterior insula and the cerebellum, please refer to Figure 1.

In another study, the mental representation of more complex, but still sublexical structures (nonwords) was examined by McGettigan et al. [113]. The study employed a phonological WM task based on the inner repetition or passive listening of nonwords. Repetition showed increased activation in the bilateral planum temporale laterally and a specific posteromedial region on the left hemisphere [113]. Increasing the loads by adding consonant clusters and syllables had a positive effect on left pre-SMA and left precentral gyrus [113]. The results from both experiments combined support the conclusions reached by Marvel et al, suggesting that premotor regions are engaged during an increased verbal WM demand [112]. Additionally, it provides more evidence for the role of the postero-medial planum temporale as a “phonological store” devoid of semantic context [113].

The interaction between the production of words (using inner speech) and WM function has been investigated [114]. Saliently generated words activated the left BA 44/45, left superior frontal gyrus, left inferior occipital gyrus and the cerebellum. Low WM scores were associated with an increased right-hemispheric laterality for language. It could be deduced that lower WM capacity could be a driving force behind right hemispheric use during language functions [114].

During language production, the transformational process from an internal representation to an outer speech involves several WM-related areas. Apart from the left IFG, a set of WM regions participate in this creation process, likely supported by an inner speech mechanism. This system relying on secondary motor areas, supports verbal WM to store and further manipulate language information. During the repetition, the planum temporale and other near regions are likely to be involved in storing phonological information and eventually linking to these secondary motor areas. Thus, the link between sensory and motor aspects of language could rely on these connections. As observed during language comprehension, involvement of the right hemisphere has been reported in individuals with lower WM capacity. However, the data presented by Powell et al. is more suggestive of a right lateralized language production being the cause and not the consequence of poor verbal WM skills [114].

### 5.3. Sentence and grammatical structures

Although single words can have a meaning on their own and can be used to convey messages (e.g., Stop!), most communication is structured into sentences. Sentences are inevitably subject to grammatical rules that impact the selection of words, their morphology, and the order they are placed within a sentence. These grammatical rules become internalized, and cognitive functions come into play to decrease the complexity or reduce the WM load.

Grammar is the study of the rules governing the composition of speech, including the fields of syntax and morphology [115]. Syntax is the combination of words into phrases and phrases into sentences. The simplest form is the declarative sentence with a structure of subject-verb-object. Morphology, on the other hand, is the combination of morphemes and words into complex words [115]. Both syntax and morphology have been investigated concerning the interaction with WM functions. Word generation can be examined as part of a verbal fluency task, usually deprived of grammatical influence [114], and cued production tasks [115]. The latter measures morphological aspects of word generation, since the word morpheme depends on its syntactic context. Syntactic sentence processing can be studied by altering argument ordering and retrieval [116]. The next section scrutinizes evidence on the interaction between grammatical composition and WM as well as the interaction between sentence structures and WM processes.

#### 5.3.1. Morphological processing

With regards to morphology, particular word inflections are usually cued by the context. To investigate the interaction between WM and morphology, during a silent word generation task, the missing word of a sentence was cued by providing the rest of the sentence an accurate context [115]. This elicited activation of the occipital cortex (perception of visual stimuli: the written sentence) and left temporal areas, including Wernicke's area. The left IFG and surrounding inferior premotor and prefrontal cortices also showed activation. The involvement of a network including the left BA 44/45, BA 47, anterior insula and SMA in morphological inflection was shown after subtracting the fMRI activity elicited from reading only [115]. This study provided novel evidence on Broca's area's involvement, along with other left premotor and prefrontal cortices in the grammatical processing of morphosyntactic features [115]. These findings are compatible with the involvement of the same regions during simpler language production tasks, possibly linked to inner speech [112],[113]. It remains to be clarified the specific differences between more basic generations of letters or nonwords with the more elaborate production of words with variable morphosyntactic.

#### 5.3.2. Syntactic processing

Syntactic processing during the construction and comprehension of sentences relies on WM for retrieval and reordering of arguments [116]. This was examined by Meyer and colleagues by altering the order and the distance between subject, verb and object [116]. Participants were presented with sentence stimuli that required reordering and retrieving arguments, thus challenging syntactic functions and WM. It was found that reordering demands relied on activation of the left pars opercularis of the IFG (BA44), while storage and retrieval demands depended on left temporo-parietal regions, namely the supramarginal gyrus [116]. These WM functions had a different temporal resolution. Activity on the temporo-parietal region occurred earlier for retrieval; reordering activity in Broca's area took around twice the time to be initiated (300–500 ms) [116].

Syntax constructs the sentence structure. The interaction between sentence structures and WM processes have been studied directly. The neural correlates of maintaining structured sentence fragments versus unstructured word collections by manipulating WM loads and rehearsal capacities (suppressing the phonological loop) were studied [117]. During the encoding process of sentence structures versus ungrammatical word strings, increased activation was seen at inferior frontal (BA 47) and anterior temporal language-related areas, also within the medial temporal lobe, including the hippocampus [117]. During the maintenance phase, reduced engagement of the left IFG, SMA, and right middle frontal gyrus (DLPFC) was observed [117]. These findings offer an insight into potential mechanisms underlying sentence superiority. Sentence structures evoke activity in core language areas supported by long-term memory regions in the temporal lobes during the encoding phase. This could reduce the load of phonological loop areas (left IFG, SMA) and the right middle frontal gyrus in the maintenance phase, as they become disengaged [117]. This would be consistent with the involvement of episodic memory systems during semantic processing in high WM capacity readers [104]. Overall, recruiting executive processes, including long-term memory, can provide an advantage when processing long, complex syntactic structures. The generation of sentences could potentially contribute to engaging this system, hence supporting WM systems to become more efficient.

All in all, studies examining the interaction between grammatical composition and WM suggest that Broca's area has a major role, not only during basic language production but also during more complex syntactic and morphological processing. Generating words with a particular syntactic morphology involves the left premotor and prefrontal cortices. On the other hand, the demand for maintaining and retrieving syntactic information relies on the left pars opercularis of the IFG (BA44). However, storage and retrieval demands depend on left temporo-parietal regions, namely the left supramarginal gyrus. Construction of sentence structures heavily engages long-term memory systems in medial temporal regions, which eases the load on core language areas and WM systems (left IFG, SMA and right DLPFC) [117].

### 5.4. Language and working memory neural networks

The study of the human nervous system at the network level is compromised by investigating different networks at both spatial and temporal scales [118]. The network is studied at two overlapping functional and structural levels [119]. The functional network is facilitated by the underlying structural connections [119]. However, in the functional examination of the brain (i.e., fMRI studies), co-activation of different regions, even without proven structural connections, is regarded as evidence supporting functional connectivity [120]. A plethora of fMRI studies investigating the neural correlates of WM involvement in linguistic processes take a network approach, as described below.

The literature hypothesizes the existence of at least three network systems in language processing [121]. Depending on the modality of the input (visual or auditory), one of the two relevant networks becomes involved before the third network's involvement, which is the core language system [16],[121]. Written speech involves areas in the visual processing network that are relevant to the processing of the visual format of the letter, the word and the sentence, as well as areas such as the left fusiform gyrus, which is suggested to be the visual word form area [16],[122]. On the other hand, spoken language activates a network that includes temporal areas such as the STG and the superior temporal sulcus [16]. In this view, the WM plays a key role in securing the accessibility of the input for further processing. WM itself is divided into two components: syntactic WM that is syntax specific and incorporates the activation of areas such as the IFG [123], and phonological WM that is related to the maintenance of language sounds, word orders and sentence structure [75],[116],[124]. In line with these findings, Newman et al. also suggest the involvement of the IFG, the DLPFC and parietal lobe structures when an individual is engaged in a task related to phonological WM and language comprehension [125]. A neuroanatomical representation of these structures is available on Figure 1.

The IFG is one of the four frontal regions suggested to be involved in all memory systems [126]. Further evidence supporting the IFG's role in the processing of phonological WM comes from Cooke and colleagues' study investigating the effect of violation of grammatical expectations on the brain's activation [127]. They suggested that the left IFG and orbitofrontal cortex (OFC), with a contribution from left temporal and anterior cingulate cortices, are involved in detecting grammatical violations. Notably, the dorsal portion of the IFG was active with grammatical violations that featured a higher WM demand [127]. Further support for the involvement of this network in the processing and comprehension of a single word comes from a recent study by Tomasi and Volkow [128]. They showed that beyond the IFG and OFC (BA 44/45 and 47), the anterior temporal cortex (BA 20 and 21) and the angular gyrus are other nodes that contribute to reading recognition and word comprehension. The latter had been previously suggested to be involved in recognition of written language forms [129],[130]. Besides, activation of the precuneus has been interpreted to reflect the engagement of attentional networks as well [128]. A previous network analysis suggested a negative correlation between the precuneus and the Broca-Wernicke network [131]. Precuneus is structurally and functionally (positively) connected to the DMN [132]. It has been shown that the DMN's activity reduces during cognitive-demanding tasks such as those related to WM [133]. For a schematic illustration of the precuneus, IFG (including Broca's area), the temporal gyri and the angular gyrus, see Figure 1.

In another effort, Mineroff and colleagues proposed functional dissociability between the core language network (left frontotemporal) and two other networks that are known to carry the main load of cognitive labor: the Multiple Demand (MD) network, which supports executive control and WM, including complex language comprehension, and the DMN, which supports introspection and is more active in the absence of external stimuli [134]. In line with Tomasi and Volkow's suggestions, activation in the MD network was associated with reducing activity within the DMN, which also did not respond to language comprehension [128]. The only region that was not completely dissociable with the language network was the left temporoparietal junction, which responded to sentences rather than nonwords [134]. It was suggested that both networks might overlap at a location near this junction and the left angular gyrus.

In conclusion, different networks have been suggested to be involved in the cognitive processes in language production and comprehension. These networks have been shown to work in relation to one another. Activation in the network suggested to be related to WM is positively associated with the core language-processing network (possibly using the left middle frontal gyrus as a hub). In turn, activation in these two networks is associated with the deactivation of the DMN.

Figure 1 illustrates the main brain areas that have been reported to be involved in tasks reflecting the interaction between language and working memory. This includes at least three networks: a core language network, the phonological loop and other WM-related areas not involved in the phonological loop. Additionally, areas reflecting individual differences in WM capacity have been highlighted as such.

## 6. Limitations and future directions

The limitations of this study are two-fold in nature. First come the limitations regarding this review. Although we followed a systematic approach to the search, this study remains a non-comprehensive review of the literature. This review is not devoid of selection biases and our search could have missed important publications. Further, it is also important to note that the conclusions are based on the authors' judgment.

Additionally, we only reported studies on healthy adults and excluded studies on children, older adults, bilingual or multilingual population, clinical cases, music, sign language, speech, motor processing, review papers, meta-analyses, EEG/ERP, and PET studies. Each of these topics can help shed light on the interaction between language and WM systems from a different perspective. However, given the specific conditions associated to each topic, a discrete review should be dedicated to discuss each topic. For example, the interaction of language and WM in children can be discussed in the context of developmental factors, and perhaps computational learning models could help understand how cognitive systems evolve and interplay in the course of development.

Another example is the language-WM interaction in the context of a disorder, both in terms of language or WM impairment and of the mechanisms developed to compensate for such impairments. The initial key word search for this review, yielded only four studies that directly assessed the interaction between language and WM in the context of a pathology. The results of studies on teenagers with specific language impairment [135] and dyslexia [136] are in line with the conclusions of this review. Thus, in both cases, the group with the condition showed hypoactivation in the left precentral sulcus (BA 6) and parietal lobe (both including BA 7), when compared to healthy participants [135],[136]. In contrast, a study on the neural substrates of verbal WM of deaf signers found an over-reliance on the left parietal regions [137]. Finally, patterns of language and WM network dysfunction were found to differ across two clinical variants of Alzheimer's disease [138]. Although both studies showed reduced connectivity in the right parietal WM networks compared to healthy controls, the one with a predominant aphasic phenotype showed significantly more disruptions in the left temporal language areas, as well as inferior parietal and prefrontal WM networks [138]. Future research on language-WM interaction in the context of disorders can contribute to better understanding of the normal processes, pathological conditions, compensation mechanisms, and may lead us to more effective treatment options.

Second, the limitations inherent in the literature on language and WM interaction. Studies with a narrow focus on this subject are limited to a small number. In addition, there are limitations associated with the choice of methodology. The first limitation concerning fMRI techniques comes with the noise produced by the MRI machine to acquire data. MRI machines make a loud noise when they are working (56–130 dB). MRI compatible headphones and earplugs reduce this noise considerably, but the noise is still above the standards we set in labs for the study of cognitive processes.

Besides, a significant part of our knowledge about cognitive processes comes from studies that employed psychophysical tasks. To study a specific function, a research group uses a task related to the particular aim of that research. Besides the variety in the type of the task, variations in the settings add to the variability between studies. Similarly, running an fMRI study adds another layer of variability. The tasks' design is required to be adapted to fit the scan (for example, the repetition time: TR) and the machine environment. Thus, it is more difficult to compare the output of those tasks between different studies.

Moreover, studies employ various tasks, not always well-defined or standardized, making generalizations even more difficult. Cognitive tasks themselves are rarely confined to a single cognitive domain, and there is a considerable amount of overlap between cognitive functions. Brain activation rarely shows a one on one relationship with a single cognitive domain, making all interpretations provisional or unconfirmed. Further, these studies included various designs in regards with control groups allowing for between-subject comparisons (two studies; [103],[114]) and control conditions in within-subject comparisons (Table 1).

Lastly, the results of some studies in this review were based on analyses considering a region of interest (ROI). Region of Interest is a set of brain areas determined by the researchers based on their hypothesis drawn from previous literature. Although analyses based on ROI can be advantageous given the strong support founded in evidence from the existing literature, it limits the chance for incidental findings, or the discovery of associated regions not previously reported. Accordingly, in comparison of findings in this review, we acknowledge this limitation. Table S1 lists the studies that have used ROI or the whole brain.

In this review of the literature on neural mechanisms of WM and language interaction, we focused on the studies that used fMRI as the brain imaging technique. Despite the limitations, fMRI gives us a good overview of different cortical and subcortical networks involved in the cognitive aspects of language processes. Considering the fact that WM and related processes do not limit to fast and reflective functions in the brain, limitations due to the lower temporal resolution in fMRI studies do not interfere with our goal in this review.

Future studies should have a meta-analytic approach to investigate the brain circuits involved in WM and language in more detail, allowing us to assess the contribution and quality of each study. To our knowledge, only one meta-analysis has been conducted on the neural correlates of verbal WM, as assessed by fMRI [92]. This study only considered studies involving visual verbal WM tasks and did not address further imbrications between language and other WM functions. Nonetheless, the main conclusions of this meta-analysis provide additional support to the conclusion drawn from the synthesis of the results reported in the studies included in this review. The meta-analysis finds evidence for involvement of attentional control systems in the right hemisphere and the caudate for response suppression [92]. The right basal ganglia, potentially collaborating with the left pre-SMA, were found to be involved in various WM processes. Additionally, load effects were primarily observed in bilateral prefrontal areas, including the DLPFC [92]. These results are consistent with the conclusion of the present study.

Further, future studies need to consider designing an experiment with better control/contrast conditions. Both language processing and WM function are modulated by attention. It would be interesting to observe the dynamic between language and WM interaction modulated by the effect of attention. Finally, an advanced computational model paradigm can reveal all contributing factors in how this dynamic interaction occurs. The complexity of the task, cognitive demand and linguistic modality are a few examples that should be factored in such a model.

## 7. Conclusion

Language is an important part of higher cognition and plays a crucial role in learning. The extensive body of behavioral and neuroimaging research on language processes brings evidence that processing different linguistic tasks and the brain regions involved in their processing can be dynamic (for reviews see [15],[139]–[141]). Language interacts bidirectionally with all higher cognitive domains, WM included. WM allows for holding the information while completing language tasks, helps with language comprehension, language production, and storage of new information into long-term memory. Thus, it would not be farfetched to speculate that WM demands would play a role in the dynamic changes of language processing. On the other hand, it is hard to exclude language from WM functions in any given task. The tasks used to study this interaction are modified WM tasks that require language comprehension (reading, listening), production (expression, mental speech), or analysis of grammatical structures.

Few studies have undertaken the task to examine the neural correlates of the interaction between WM and Language. Although the reading span task seems to be the most popular approach to engage both systems, studies have used different tasks, some of them non-standardized for the study of WM (refer to Table 1). This makes it very difficult to draw inferences about specific and common activation patterns. Nonetheless, the literature reviewed shows that language-based tasks that involve WM engage the activation of a few common neural systems.

Extensive evidence suggested that the phonological loop (Broca-Wernicke), involved in maintaining and retrieval during verbal WM, is also essential for language processing at multiple levels (e.g., ambiguity processing, reordering words). Activation of the left IFG was also found to correlate with high WM capacity in syntactic complexity. There is a clear dynamic interaction between WM and Language, which is levered by cognitive demand: task complexity can moderate the involvement of WM in language processing. Complex linguistics leads to the recruitment of core WM areas in the prefrontal cortex (including the DLPFC) and right hemispheric regions linked to the visuospatial sketchpad. Further involvement of subcortical structures, particularly the basal ganglia (caudate), but also premotor areas (pre-SMA, SMA, cerebellum), have been reported associated with the processing of high complexity linguistic components (particularly syntactic). Figure 1 illustrates the main brain areas that have been reported to be involved in tasks reflecting the interaction between language and working memory.

The dynamics of language and WM interaction are also evidenced by the correlations observed between low and high WM capacity individuals (as mostly measured by the reading span task) with areas involved in verbal and non-verbal WM. Low WM capacity induces the activation of similar regions as those activated in response to increased language complexity, while high WM capacity readers engage more buffer memory systems (medial temporal lobe and other midline cortical areas) to decrease the WM demands on limited resources. This strategy is also observed during the maintenance of sentence structures, allowing for more efficient encoding, also described as “chunking” by Cowan in the WM literature.

Different networks have been suggested to be involved in language-WM interactions. The differences reflect language modality and the involvement of other cognitive processes such as attention. It would be necessary to further investigate the level of activation of the separate functions and in relation to each other, in order to improve the efficiency of the brain's function.

## Footnotes

1 The n-back task is administered broadly to study WM. The task consists of a list of visual or auditory stimuli that is presented to participants. They are instructed to indicate whether each stimulus is a correct match with the *n*^th^ stimulus presented before. The n-back task demands constantly storing, updating information and inhibiting distractors. The cognitive load can be altered in this task by changing the value of *n*, which correspondingly may affect accuracy and reaction times. In the n-back task, visual or auditory stimuli are stored in the phonological loop or visuospatial sketchpad loop while selecting the correct answer depends on the central executive function. The central executive function is responsible for updating information and inhibiting the processing of irrelevant information. Brain functions linked to different types of WM (such as visuospatial WM) can be examined by using n-back tasks.

2 The WM span tasks are divided into two main categories: simple and complex span tasks. The simple span task requires encoding a list of items (e.g., words) and maintaining that information during short delay periods and then recalling them. On the other hand, the complex span task includes presenting a list of items and asking participants to memorize them while performing another cognitive task (e.g., mathematics problem-solving, reading) and then recalling them. Performing the distractive task while maintaining task-relevant information makes the maintaining phase more difficult by directing attention towards task-irrelevant information. The span tasks, particularly complex span tasks, need storing, updating information and inhibiting distractors.
