# Supplementary material for: The interaction between language and working memory: a systematic review of fMRI studies in the past two decades
Source: AIMS Neurosci. 2020 Nov 16;8(1):1–32. doi: 10.3934/Neuroscience.2021001 (PMC7815476; doi:10.3934/Neuroscience.2021001)
Supplement: Supplementary file 1 [file neurosci-08-01-001-s001.pdf]

AIMS Neuroscience

AIMS Neuroscience, 8(1): 1–32.
DOI: 10.3934/Neuroscience.2021001
Received: 03 September 2020
Accepted: 10 November 2020
Published: 16 November 2020

http://www.aimspress.com/journal/aimsph

Review

The interaction between language and working memory: a systematic review of fMRI studies in the past two decades

Zoha Deldar[1,5,†], Carlos Gevers-Montoro[1,2,†], Ali Khatibi[3,4] and Ladan Ghazi-Saidi[5,*]

[1] Department of Anatomy, Université du Québec à Trois-Rivières, Trois-Rivières, QC, Canada
[2] Madrid College of Chiropractic, Real Centro Universitario María Cristina, San Lorenzo de El Escorial, Madrid, Spain
[3] Centre of Precision Rehabilitation for Spinal Pain, University of Birmingham, Birmingham, UK
[4] Centre for Human Brain Health, University of Birmingham, Birmingham, UK
[5] Language and Cognition Laboratory, Department of Communication Disorders, College of Education, University of Nebraska at Kearney, USA

† Zoha Deldar and Carlos Gevers-Montoro contributed equally to this work.

* **Correspondence:** Email: ghazisaidil2@unk.edu.

Table S1. A summary of the magnetic resonance imaging approach and analysis technique employed in the 20 studies included in this review.

Authors (year)	MR Method and Analysis		MRI Analysis
	MR field strength	Analysis software	
Buchweitz et al. (2009) [16]	3 T	SPM2	Whole-brain
Moore et al. (2013) [72]	3 T	AFNI	Whole-brain and functional connectivity
Buchweitz et al. (2012) [97]	3 T	SPM2	Regions of Interest and functional Connectivity
Fiebach et al. (2004) [98]	3 T	LIPSIA and SPM99	Whole-brain
Mason & Just (2007) [99]	1.5 T	FIASCO and SPM99	Whole-brain
Rudner et al. (2013) [102]	3 T	SPM8	Whole-brain
Rudner et al. (2005) [103]	1.5 T	SPM99	Whole-brain
Newman et al. (2013) [104]	3 T	SPM8	Functional connectivity
Wallentin et al. (2006) [110]	3 T	SPM2	Whole-brain
Marvel & Desmond (2012) [112]	3 T	SPM2	Whole-brain and region of interest
McGettigan et al. (2011) [113]	1.5 T	SPM5	Regions of Interest
Powell et al. (2012) [114]	3 T	SPM5	Whole-brain
Sahin et al. (2006) [115]	3 T	FS-FAST	Whole-brain and region of interest
Meyer et al. (2012) [116]	3 T	SPM8	Whole-brain
Bonhage et al. (2014) [117]	3 T	SPM8	Whole-brain
Makuuchi & Friederici (2013) [121]	3 T	SPM8	Regions of Interest and functional Connectivity
Newman et al. (2002) [125]	1.5 T	FIASCO	Regions of Interest and functional Connectivity
Cooke et al. (2006) [127]	1.5 T	SPM99	Regions of Interest and functional Connectivity
Tomasi & Volkow (2020) [128]	3 T	IDL	Functional connectivity
Mineroff et al. (2018) [134]	3 T	SPM5	Regions of Interest and functional Connectivity

AIMS Press

© 2021 the Author(s), licensee AIMS Press. This is an open access article distributed under the terms of the Creative Commons Attribution License (http://creativecommons.org/licenses/by/4.0)
